# Improving Insurance Protection for Rare Diseases: Economic Burden and Policy Effects — Simulation of People With Pompe Disease in China

**DOI:** 10.34172/ijhpm.2022.6282

**Published:** 2022-10-24

**Authors:** Shanquan Chen, Dong Dong

**Affiliations:** ^1^School of Clinical Medicine, University of Cambridge, Cambridge, UK.; ^2^Jockey Club School of Public Health and Primary Care, Chinese University of Hong Kong, Hong Kong, China.; ^3^Shenzhen Research Institute, The Chinese University of Hong Kong, Shenzhen, China.

**Keywords:** Pompe Disease, Economic Burden, Catastrophic Health Expenditure, Impoverishment, Policy Simulation, China

## Abstract

**Background:** The economic burden of Pompe disease (PD) is under-researched. This study aimed to fill this gap and provide evidence-based suggestions for policy improvement based on policy simulation.

**Methods:** Data were derived from a nationally based cross-sectional survey on rare diseases in early 2018. Answers from 92 PD patients were used for data analysis and simulation. Catastrophic health expenditure (CHE) and impoverishment due to illness (IDI) were adopted to measure PD patients’ economic burden. Two typical reimbursement patterns, a dosage-based model and a cost-based model, in China were simulated.

**Results:** Twenty-four pediatric and 68 adult PD patients were investigated. Families with pediatric PD patients on average had lower annual household incomes than families with adult PD patients (RMB 37 890 vs. RMB 66 120). The direct medical expense and out-of-pocket expenses were almost double for pediatric patients compared with adult patients (RMB 120 050 vs. RMB 66 350; RMB 112 710 vs. RMB 57 940, respectively). The direct non-medical expense for patients was almost six times the expense of adult patients (RMB 73 790 vs. RMB 13 080, respectively). About 88.24% of families with pediatric PD patients and 67.21% of families with adult PD patients suffered from CHE. Around 84.21% of families with pediatric PD patients and 45.90% of families with adult PD patients were forced to live in poverty due to illness. The simulation indicated that, although the two current reimbursement schemes helped reduce CHE, they almost had no effect on reducing IDI; the dosage-based model was more sensitive to changes in policy parameters.

**Conclusion:** Our study highlighted the alarmingly high disease burden faced by PD patients with first-hand patient-reported evidence. Our series of simulations could be a good reference for China and other countries to improve their reimbursement policy regarding PD.

## Background

**Key Messages**

** Implications for policy makers**

- Pompe disease (PD), also known as glycogen storage disease type II, is a rare autosomal recessive disorder. Medical treatment for PD is available, yet its high price has imposed a huge economic burden on patients and their families.
- Insufficient or even lack of medical insurance has caused many PD patients and their families to suffer from catastrophic health expenditures (CHEs) and impoverishment due to illness (IDI). These situations are inconsistent with one of the Sustainable Development Goals: Universal Health Coverage for all.
- In China, dosage-based and cost-based models are used as two pilot customized medical insurance options for PD patients. Globally, there are also other options, such as fully coverage by universal healthcare or special schemes for rare diseases (eg, in Taiwan or Australia).
- Customized medical insurance is needed for PD patients, and the dosage-based model is more feasible and has more potential for further development than the cost-based model.

** Implications for the public**

While this research focuses on the perspective of medical insurance and not directly on the patients, the findings demonstrate a need for customized medical insurance for Pompe disease (PD) patients to relieve their economic burden, especially under the context that little evidence is available about the cost burden on PD patients. Our study enriches corresponding evidence and helps other researchers estimate the potential economic benefit of treatment. Additionally, our study also provides suggestions for countries that want to improve their policies to cover PD. Our simulation suggests that compared with cost-based strategies, dosage-based ones have more potential to reduce the economic burden of PD patients. Our study also recommends that the special needs of pediatric PD patients compared to adult PD patients should be taken into consideration when designing local policies.

Pompe disease (PD), also known as glycogen storage disease type II, is a rare, multisystemic, hereditary disease, which is caused by pathogenic variations in the *GAA* gene.^1,2^ The *GAA* gene contains the genetic information for the production and function of a protein called acid alpha-glucosidase. PD is generally classified into two forms: (1) infantile onset PD (IOPD), when the disease presents during the first year of life; and (2) late onset PD (LOPD), when it presents afterward, including childhood, juvenile, and adult onset.^3^ Major symptoms and body dysfunctions caused by PD result from the accumulation of glycogen in the skeletal muscles, heart, liver, and nervous system, which often first lead to disabilities and later to death.^4^ The infantile form of PD usually causes the death of a newborn within the first year of her or his birth. For patients with LOPD, their mobility and respiratory systems are severely affected.^5-7^ Based on a formula in a previous publication,^8^ 231 newborns suffered IOPD and 55 991 individuals suffered LOPD in China in 2020. As of the end of 2021, PD has not been included in newborn screening in China as it is in some countries, such as the United States.^9^

 Currently, the only specific treatment for PD available worldwide is alglucosidase alfa (Myozyme^®^), a type of enzyme replacement therapy (ERT). This therapy is a life-long treatment and requires regular intervention. Myozyme^®^ is indicated in adults and pediatric patients of all ages, and the sooner a patient starts treatment with Myozyme^®^, the better the results.^10^ The recommended dose regimen of Myozyme^®^ is 20 mg/kg of body weight administered once every two weeks. Myozyme^®^ was approved as ERT treatment for PD by Europe, the Middle East, and Africa and the US Food and Drug Administration in 2006 and was introduced to China in 2017. However, until the end of 2021, except for a few cities and provinces, it has not been included in the national drug reimbursement list or most provincial lists. Like many other orphan drug products, the price of Myozyme^®^ is extremely high, and only a few families can afford it. It was estimated that in 2018 the average annual cost of Myozyme^®^ for an adult PD patient was approximately RMB 3 000 000, more than forty times the average annual household income of a Chinese family.^1,11^

 ERT for PD is not only expensive in China. In Europe, its average cost in 2011 was about EUR 300 000 (approximately RMB 2 360 000) per patient per year.^1^ However, what makes the burden of ERT greater in China is that almost all PD patients have to pay out of pocket, and there is almost no insurance coverage, either from the government or from the market, to help them alleviate the cost. As a result, Chinese PD patients are fully exposed to the high economic burden brought about by the disease. This situation is inconsistent with one of the Sustainable Development Goals defined by the United Nations in January 2016^12^ and the advocated goal of undergoing Chinese health reform since 2009; that is, achieving universal health coverage for all.^13^

 We selected PD from a group of rare diseases with ultra-expensive treatments to highlight three main problems of healthcare. First, considerable underuse of medicine in China. By the end of 2017, among the 61 registered PD patients of a representative organization in China, only 12 (19.7%) used Myozyme^®^, with only 4 (6.6%) patients using the medicine continuously.^14^ From 2014 to 2018, according to the same organization, 14 pediatric PD patients died and 11 of them had never used the drug because of the unaffordable price.^14^ By contrast, 100% of the IOPD (9/9) and 97% of the LOPD patients (34/35) in Japan screened during April 2018 and March 2019,^15^ 98% of the LOPD patients (51/52) in Belgium screened during 2010 and 2017,^16^ and 70% of the LOPD patients (7/10) in Poland screened during June 2014 and May 2017^17^ received the Myozyme^®^ treatment.

 Second, even though PD patients have borne a huge economic burden, there is no single study that has scientifically and thoroughly investigated this issue in China, neither has it been collectively studied as a critical issue faced by many Chinese rare disease patients. Special attention to PD is relatively scarce, even internationally. Hence, the primary aim of this study is to fill such gaps.

 Third, while having relatively low prevalence numbers and being only one of the 7000-8000 rare diseases in the world, PD is among the 5% of all rare diseases that have specific treatments. Many medicines for rare diseases are labelled as orphan drugs that often imply high prices, a small market, and a heavy economic burden. The problem of their immense economic burden is an issue that should not be overlooked. Fortunately, several cities in China have implemented pilot reimbursement policies for PD patients. The two most typical reimbursement patterns are the dosage-based reimbursement policy in Tianjin, and the cost-based reimbursement policy in Jining. A simulation of the effect of these piloted models on the economic burden is necessary. Corresponding results could provide evidence-based suggestions for policy improvements, both for PD but also for other rare diseases.

###  Two Pilot Reimbursement Schemes in China 

 Two reimbursement schemes were piloted in Tianjin and Jining, China, respectively. Because of the localized management of patients at the city or provincial level, patients in China can only apply for medical reimbursement via local insurance schemes available in the city or province where they have registered residency or have an official employment. The two pilot schemes both offer PD-specific reimbursements and are an extension of the basic medical insurance (BMI), which is provided by the government and covers both urban and rural residents. More specifically, for PD patients who need to claim expenses spent on Myozyme^®^, they will go through the pilot scheme rather than the BMI for diseases other than PD. To be noted, the PD patients sampled in this study did not live in Tianjin and Jining and were not covered by these two pilot schemes. Therefore, we will simulate the results based on the details of these two typical reimbursement schemes to explore to what extent both pilot schemes reduce the economic burden of families with PD patients, under the assumption that they were covered by the pilot policies. Details of these two typical reimbursement schemes are as follows:

####  (1) The Dosage-Based Model

 The reimbursement policy in Tianjin is based on the dosage of medicine (Myozyme^®^) used per patient. All PD patients who use Myozyme^®^ for treatment can benefit from the policy. There is no reimbursement deductible line, and patients need to apply for reimbursement on a monthly basis. Each month, patients have a 70% reimbursement rate for the first five vials of Myozyme^®^ used. For the next five vials of Myozyme^®^ (up to a total of 10 vials), 60% of the total costs can be reimbursed; for the third batch of vials (up to a total of 15 vials), the reimbursement rate is 50%; no reimbursement is provided for the 16^th^ vials and afterwards. For instance, if one PD patient injects 14 vials of Myozyme^®^ in one month, and if the cost of Myozyme^®^ is RMB 5645 per vial, after reimbursement from the medical insurance, the patient still needs to pay at least RMB 31 047.5 per month calculated as follows:

*(1) Total cost=14 vials × 5645 RMB/vials=79030 RMB*

*(2) Reimbursement for 0-5th vials=5 vials × 5645 RMB/vials × 0.70=19757.5 RMB*

*(3) Reimbursement for 6th-10th vials=5 vials × 5645 RMB/vials × 0.60= 16,935 RMB*

*(4) Reimbursement for >10th vials=4 vials × 5645 RMB/vials ×0.50=11290 RMB*

*(5) Actual self paid cost=(1)-(2)-(3)-(4)= 79030-19757.5-16935-11290=31047.5 RMB*

####  (2) The Cost-Based Model

 The reimbursement policy in Jining is designed based on actual medication costs. The cost of Myozyme^®^ in Jining is divided into two parts: cost not covered by insurance (deductible percentage, accounting for 30%) and cost covered by insurance (accounting for 70%). The former part is the cost paid by patients themselves. For the later part, BMI offers reimbursement on 70% of the insured cost (co-payment percentage), and the rest is covered by another special insurance, called the critical illness insurance (one type of commercial insurance [CI]), with a deductible line of RMB 12 000 and a reimbursement rate at 50%. Both BMI and CI have an annual ceiling line of RMB 150 000 and RMB 500 000, respectively. In addition, if the annual accumulated amount claimed from the CI exceeds RMB 100 000, the reimbursement rate of CI will increase to 60%. For instance, using the same example as under the dosage-based model, if a PD patient uses 14 vials of Myozyme^®^ in one month, this patient will need to pay RMB 38 008 per month out of pocket calculated as the following:

*(1) Total cost=14 vials ×5645 RMB/vials=79 030 RMB*

*(2) Cost covered by BMI=(1)×(1-0.3)=55 321 RMB*

*(3) Reimbursement from BMI=(2)× 0.70=38 724.7 RMB*

*(4) Cost covered by CI=(1)×(1-0.3)×(1-0.7)-12 000=4596.3 RMB*

*(5) Reimbursement from CI=(4)× 0.50=2298.15 RMB*

*(6) Actual self paid cost=(1)-(3)-(5)=79030-38724.7-2298.15=38007.15RMB*

 In this study, our primary objective was to evaluate the disease economic burden of PD patients using nationwide cross-sectional data from China. Our second objective was to simulate the policy effect of the two pilot reimbursement schemes (dosage-based model and cost-based model) on the economic burden of sampled PD patients.

## Methods

###  Research Subjects

 Although PD includes two subtypes, IOPD and LOPD, the population affected can actually be divided into three subgroups: infants, children, and adults.^1^ Infant PD patients aged under 1 year were not captured by our survey. In this study, we only focus on the other two subgroups: pediatric PD patients (1-18 years of age) and adult PD patients (>18 years of age).

###  Sampling Methods

 Data used for this research is derived from a large, nationwide, cross-sectional survey of people affected by rare diseases in China in 2018.^18,19^ The population information on people affected by rare diseases in China is largely unknown, and thus, no complete sample frame exists. Therefore, a non-probability, convenience sampling method was used to recruit participants. In collaboration with several national rare disease patient organizations, such as the Illness Challenge Foundation and the China Pompe Care Center (the only national PD patient organizations in China), information about the survey was distributed via their online and offline patient networks. Recruitment information was also shared by individual patients within the organizations through snowball sampling.

###  Survey Procedure and Recruitment of Pompe Disease Patients

 The survey was conducted primarily online to maximize accessibility for the dispersed population. The survey was self-administered. Previous studies have shown that results from this type of survey can be much more accurate than surveys via telephone.^20,21^ In this case, participants followed a link to the survey website and informed consent was obtained before they were exposed to the questionnaire. At the beginning of the questionnaire, a series of questions was used to identify the target respondents of the survey (ie, people affected by rare diseases in China). Patients under the age of 18 were instructed to stop the survey and refer the survey link to their legal guardians. Main caregivers and patients were identified and diverted to two different versions of the questionnaires (which covered the same measures, but the questions were formed in different ways to obtain more accurate answers). The survey was conducted from January 1 to February 15, 2018.

 In total, responses from 92 PD patients were collected from the 2018 survey. Although small in number, the 92 PD patients who participated in the survey constituted all PD patients with a definite clinical diagnosis at the time of the survey. In other words, these 92 patients were the largest possible sample of diagnosed PD patients who could be reached in China with non-probability and convenience sampling by February 15, 2018. In this sense, the sample was not a “sample” but a complete set (or “population”) of all possible observations of Chinese PD patients at that time. The same patient data set was used in previous publications on quality of life^8^ and difficulties in accessing a definitive diagnosis of a rare disease.^19^

###  Outcomes of Interest and Measurement

 In this study, two commonly used indicators, frequently emphasized in the United Nations’ Sustainable Development Goals and healthcare policy-making,^22^ were adopted to measure PD patients’ economic burden; that is, catastrophic health expenditure (CHE) and impoverishment due to illness (IDI).

- *CHE*: when the cost of treatment for a disease exceeds a certain percentage of income, the disease is considered to cause catastrophic expenditures for the patient. There is no commonly accepted criterion for defining CHE.^23^ The criteria vary from 10% of income,^24,25^ 10% of household consumption^26^ to 40% of disposable income.^27,28^ In this study, we regarded out-of-pocket health expenditures exceeding 10% of annual family income as potentially CHE, as it suits the Chinese context better. The other two are more sensitive to people’s behaviour of saving and consuming. The Chinese have a culture of precautionary savings and are more inclined to avoid unnecessary consumption; whereas, they are relatively generous when they need to spend on medical expenses for their loved ones.^29,30^ Hence the 10% of family income criterion was adopted.
- *IDI*: when the household income level per capita falls below the poverty line because of the cost of disease treatment, the disease can be considered to cause impoverishment to the patient’s family.^31^ In this study, the poverty line is based on the criterion set up by the World Bank, namely, $US 1.90 per person per day, which is about RMB 4978 per person per year recalculated with the exchange rate applicable during the survey period.

Other related variables and their definitions are as follows:

- *Household income* was collected on the basis of the entire family and included the aggregate of household income from production, wage income of household members, transfer income (remittances, welfare), and property income (interest, rent).
- *Direct medical expense* was collected for the patient only and included outpatient expenses, hospitalization expenses, and self-purchased drugs or equipment costs due to medical treatment.
- *Direct non-medical expense* was collected for the entire family involved in the process of seeking diagnosis and treatment for the PD patient and included travel, lodging, and food costs.
- *BMI* specifically refers to the three BMI plans sponsored by the Chinese government, including Urban Employees Basic Medical Insurance, Urban Residents’ BMI, and New Rural Cooperative Medical Care.
- *CI* includes medical insurance for major illnesses provided by the government via a public-private collaborative scheme, such as critical illness insurance, or private medical insurance purchased by patients.
- *Out-of-pocket payment* refers to the medical expenses incurred by the individual patients after the reimbursement of BMI and CI.

All of the above data were collected through a bottom-up approach using two steps. We followed the CHARLS, a national survey in China, to design our bottom-up approach by considering the structure and characteristics of China’s health service system.^32^ In this approach, the outpatient expenses were collected by the level of the health institution visited (primary care, secondary care, or tertiary care); the hospitalization expenses were collected by the number of times the patient was admitted to an inpatient stay; the cost of self-purchased drugs or equipment was collected by the place of purchase (town, county, city, or other cities). Briefly, we asked patients or their caregivers the number of days or months they used for earning, and the frequency or times of pursuing healthcare for PD during the last twelve months. The second step was to estimate the income per day or per month per income source and the cost per time of pursuing healthcare for PD. The total costs and income was derived through multiplication and summing. A detailed process of the bottom-up approach is provided in Figures S1-S4 (Supplementary file 1).

###  Statistical Analysis and Policy Effect Simulation

 To describe the baseline characteristics of this cohort, category variables are reported as a number (percentage), and continuous variables are reported as a mean (standard deviation).

 To comprehend the source of economic burden for PD patients in China, and to know how their economic burden was influenced by the insurance they had, the economic burden of PD patients was estimated in a stepwise way as follows: Step 1 - the economic burden assuming they paid all the direct medial costs by themselves; Step 2 - the economic burden after they benefited from BMI; Step 3 - the economic burden after they benefited from the CI; and Step 4 - the economic burden after including the direct non-medical costs.

 To know how the pilot reimbursement polices influenced PD patients’ economic burden, we simulated the results by assuming that the patients were covered by the policies. As this study will reveal, the primary economic burden of PD patients came from direct medical costs. Previous studies also demonstrated that Myozyme^®^ users, especially adult patients, did not use the full dose.^1^ Hence, in this study, the policy effect simulation was primarily based on direct medical costs, which was calculated using the following assumptions:

- Only Myozyme^®^ cost was taken into consideration, regardless of other medications that the patients might have taken.
- As for the dosage of Myozyme^®^, we assumed paediatric patients would take the full dosage, whereas adult patients would take ⅛ to ¼ of the full dosage, which is what many PD patients in China do in reality. Here, full dosage means the dosage prescribed by physicians based on the recommendation of the manufacturer.

Additional simulation constraints are based on clinical guidance and actual practice in China:

- The Myozyme^®^ dosage is based on patients’ weight; ie, 20 mg/kg.
- PD patients take Myozyme^®^ twice a month.
- The unit of Myozyme^®^ is 50 mg/vial.
- The list price of Myozyme^®^ in our data collection period (January 1 to February 15, 2018) is RMB 5645/vial.

In addition to the simulation of the two pilot policies, we also made series of simulations based on variant policy parameters (eg, the co-payment percentage) to offer more useful suggestions on policy improvement. The full policy parameters used in the simulation are listed in Table 1. Due to the small simple size (although not small relative to the low prevalence of PD), we adopted bootstrapping to enhance the robustness of estimations. Bootstrapping is a statistical procedure, which generates sub-datasets by repeatedly drawing random samples from the original data with replacement, then generates estimations for each sub-dataset, and then calculates the final estimation based on the above series of estimations.^33^

**Table 1:** Parameters Used in Simulation

**Parameters**	**Value**	**Examples**
**Commonly shared parameters for both dosage-based and cost-based models**		
Standard of Myozyme® prescription	20 mg/kg	
Frequency of taking Myozyme®	2 time per month	
Unit of Myozyme®	50 mg/vial	
Price of Myozyme®	5645 RMB/vial	
Adherence to prescription	Pediatric patients take the full clinically suggested dosage, whereas adult patients take ⅛ to ¼ of the clinically suggested dosage	
**Specific parameters for the dosage-based model**		
Reimbursement rate	*Three piecewise reimbursement*Piloted plan: "70-60-50" Variant plans: "80-70-60", "90-80-70", "100-90-80" *Four piecewise reimbursement* Variant plans: "40-30-20-10", "50-40-30-20", "60-50-40-30", "70-60-50-40", "80-70-60-50", "90-80-70-60", "100-90-80-70"	“70-60-50” represents a reimbursement plan with three piecewise reimbursements, indicating that patients have a 70% reimbursement ratio for 1-5 vials of Myozyme®, 60% reimbursement ratio for vials 6 to 10 of Myozyme®, 50% reimbursement ratio for vials 10 to 15 of Myozyme®, and no reimbursement for vials 16 and above. “70-60-50” is the piloted plan, and others are variant plans. “40-30-20-10” represents a reimbursement plan with four piecewise reimbursements, indicating that patients have a 40% reimbursement ratio for 1-5 vials of Myozyme®, 30% reimbursement ratio for vials 6 to 10 of Myozyme®, 20% reimbursement ratio for vials 10 to 15 of Myozyme®, 10% reimbursement ratio for vials 16 to 20 of Myozyme®, and no reimbursement for vials 21 and above.
**Specific parameters for the cost-based model**		
Part of BMI		Take the pilot plan as an example. The cost of Myozyme® is divided into two parts: cost not covered by insurance (deductible percentage, accounting for 30%) and cost covered by insurance (accounting for 70%). The former part is the cost paid by patients themselves. For the later part, the BMI offers reimbursement on 70% of the insured cost (co-payment percentage of BMI), and the rest is covered by another special insurance, a type of CI, with a deductible line of RMB 12 000 and a reimbursement rate of 50% (co-payment percentage of CI (1)). Both BMI and CI have an annual ceiling of RMB 150 000 and RMB 500 000, respectively. In addition, if the annual accumulated amount claimed from the CI exceeds RMB 100 000, the reimbursement rate of CI will increase to 60% (co-payment percentage of CI (2)).
Deductible percentage	Pilot plan: 30% Variant plans: 20%, 10%.	
Co-payment percentage of BMI	Pilot plan: 70% Variant plans: 80%, 90%, 100%	
Ceiling of BMI	Pilot plan: 150 000 RMB Variant plans: 175 000 RMB, 200 000 RMB	
Part of CI		
Co-payment percentage of CI (1)	Pilot plan: 50% Variant plans: 60%, 70%, 80%, 90%	
Ceiling of CI (1)	Pilot plan: 100 000 RMB Variant plans: 50 000 RMB, 75 000 RMB, 125 000 RMB	
Co-payment percentage of CI (2)	Co-payment percentage of CI (1) + 10%	
Ceiling of CI (2)	Piloted plan: 500,000 RMB Variant plans: 400 000 RMB, 425 000 RMB, 450 000 RMB, 475 000 RMB, 525 000 RMB, 550 000 RMB, 575 000 RMB, 600 000 RMB	

Abbreviations: BMI, basic medical insurance; CI, commercial insurance.

 All data analysis and simulation were done in R (3.6.0).^34^

## Results

###  Basic Characteristic of Pompe Disease Patients

 In this study, a total of 92 PD patients, all of whom have LOPD were investigated (Table 2). Approximately a quarter of the sampled PD patients are children, whose average age is 5.9 years, compared with 30.0 years for the adults. The percentage of male patients is higher in pediatric PD patients than the adult patients (62.5% vs. 47.1%). The ethnicity is almost the same in both pediatric and adult PD patients, with about 96% of both populations being of Han ethnicity. The urban/rural ratio shows a balance in adult patients (50.0% vs. 48.5%), but seven out of ten pediatric patients live in rural areas. Compared with adult patients, pediatric patients went through shorter time intervals (1.3 vs. 4.3 years) from symptom onset to having a definitive diagnosis and visited fewer hospitals (2.7 vs. 3.9) and fewer departments (2.4 vs. 3.1) during this process. Families with pediatric PD patients on average have lower annual household income than families with adult PD patients (RMB 37 890 vs. RMB 66 120). The direct medical expenses and out-of-pocket expenses are almost doubled for pediatric patients compared with adult patients (RMB 120 050 vs. RMB 66 350; RMB 112 710 vs. RMB 57 940, respectively). The direct non-medical expenses for pediatric patients are almost six times that of adult patients (RMB 73 790 vs. RMB 13 080, respectively). On average, the sum of direct medical and non-medical expenses are 3.7 times the annual household income. More specifically, such expenses are 6.5 times higher than the annual household income of families with pediatric PD patients and 2.5 times the annual household income of families with adult PD patients. Pediatric patients on average would benefit from RMB 7250.4 in reimbursement from BMI and RMB 83.6 om reimbursement from CI; whereas adult patients on average would benefit from RMB 8415.59 and RMB 29.41, respectively. The accessibility of PD-related information to patients or their caregiver is low for both subgroups, with 83.3% of caregivers of pediatric PD and 95.6% of adult PD or their caregivers subjectively having difficulty accessing such information.

**Table 2:** Basic Characteristics of Pompe Disease Patients

	**Pediatric PD (n = 24)**	**Adult PD (n = 68)**
Gender (male)	15 (62.5%)	32 (47.1%)
Age	5.9 (5.6)	30 (7.4)
Ethnicity (Han)	23 (95.8%)	65 (95.6%)
Residence place		
Urban	7 (29.2%)	34 (50%)
Rural	17 (70.8%)	33 (48.5%)
Others (eg, overseas passport)		1 (1.5%)
Education		
Kindergarten/no schooling	16 (66.7%)	1 (1.5%)
Primary school	4 (16.7%)	4 (5.9%)
Middle school	3 (12.5%)	15 (22.1%)
High school	1 (4.2%)	23 (33.8%)
Three-year college degree	0 (0%)	11 (16.2%)
Bachelor’s degree or above	0 (0%)	14 (20.6%)
Employed (yes)	Not applicable	14 (20.6%)
Diagnostic experience		
Time from symptom onset to diagnosis (year)	1.3 (2.8)	4.3 (5.0)
Number of hospitals visited	2.7 (1.7)	3.9 (2.8)
Number of clinical departments visited	2.4 (1.7)	3.1 (2.0)
Annual household income		
(1000 RMB)	37.9 (32.4)	66.1 (103.5)
(1000 US dollar)	5.3 (4.5)	9.2 (14.4)
(1000 EUR)	4.9 (4.2)	8.5 (13.3)
Direct medical expense during past year		
Total cost		
(1000 RMB)	120.1 (231.6)	66.4 (245.2)
(1000 US dollar)	16.7 (32.3)	9.2 (34.2)
(1000 EUR)	15.4 (29.7)	8.5 (31.4)
Reimbursed by BMI		
(1000 RMB)	7.3 (10.9)	8.4 (21.6)
(1000 US dollar)	1.0 (1.5)	1.2 (3.0)
(1000 EUR)	0.9 (1.4)	1.1 (2.8)
Reimbursed by CI		
(1000 RMB)	0.1 (0.4)	0.03 (0.2)
(1000 US dollar)	0.01 (0.1)	0.004 (0.03)
(1000 EUR)	0.01 (0.1)	0.004 (0.03)
Out-of-pocket		
(1000 RMB)	112.7 (232.8)	57.9 (243.8)
(1000 US dollar)	15.7 (32.4)	8.1 (34.0)
(1000 EUR)	14.4 (29.8)	7.4 (31.2)
Direct non-medical expenses during the past year		
(1000 RMB)	73.8 (152.2)	13.1 (37.6)
(1000 US dollar)	10.3 (21.2)	1.8 (5.2)
(1000 EUR)	9.5 (19.5)	1.7 (4.8)
Subjective assessment on accessibility to PD related information		
Easy	0 (0%)	1 (1.5%)
General	4 (16.7%)	2 (2.9%)
Hard	20 (83.3%)	65 (95.6%)

Abbreviations: BMI, basic medical insurance; CI, commercial insurance; PD, Pompe disease.

###  Disease Economic Burden for Pompe Disease Patients

 Around 95.8% of families with pediatric patients and 67.7% of families with adult patients suffer from CHE (Table 3). This percentage decreased to 87.5% and 61.8%, respectively, after the PD patients received reimbursement from BMI, and have stayed the same after reimbursement from CI. If the direct non-medical costs are taken into consideration, the same proportion of families with pediatric patients (87.5%) and an increased number of families with adult patients (67.7%) remain left with CHE.

**Table 3:** Disease Economic Burden of Pompe Disease Patients

	**Pediatric PD**		**Adult PD**	
	**n/N**	**%**	**n/N**	**%**
CHE				
Only consider direct medical expense	23/24	95.8	46/68	67.7
Above – reimbursement by BMI	21/24	87.5	42/68	61.8
Above – reimbursement by CI	21/24	87.5	42/68	61.8
Above + direct non-medical expense	21/24	87.5	46/68	67.7
IDI				
Only consider annual household income	4/24	16.7	12/68	17.6
Above – direct medical expense	20/24	83.3	30/68	44.1
Above + reimbursement by BMI	19/24	79.2	29/68	42.7
Above + reimbursement by CI	19/24	79.2	29/68	42.7
Above – direct non-medical expense	20/24	83.3	31/68	45.6

Abbreviations: BMI, basic medical insurance; CI, commercial insurance; PD, Pompe disease; CHE, catastrophic health expenditure; IDI, impoverishment due to illness.

 About 16.7% of families with pediatric PD patients and 17.6% of families with an adult patient already were living in poverty even before they sought ERT treatment (Table 3). These ratios soar to 83.3% and 44.1%, respectively, due to the direct medical costs. The proportion of families living below the poverty line slightly decreased to 79.2% and 42.7%, respectively, as a result of the reimbursement provided by BMI; however, this proportion remained the same after reimbursement from CI. Finally, if direct non-medical costs are taken into consideration, more families with pediatric PD patients (83.3%) or adult PD patients (45.6%) are forced to live in poverty.

###  Policy Effect Simulation

**Figure 1:**
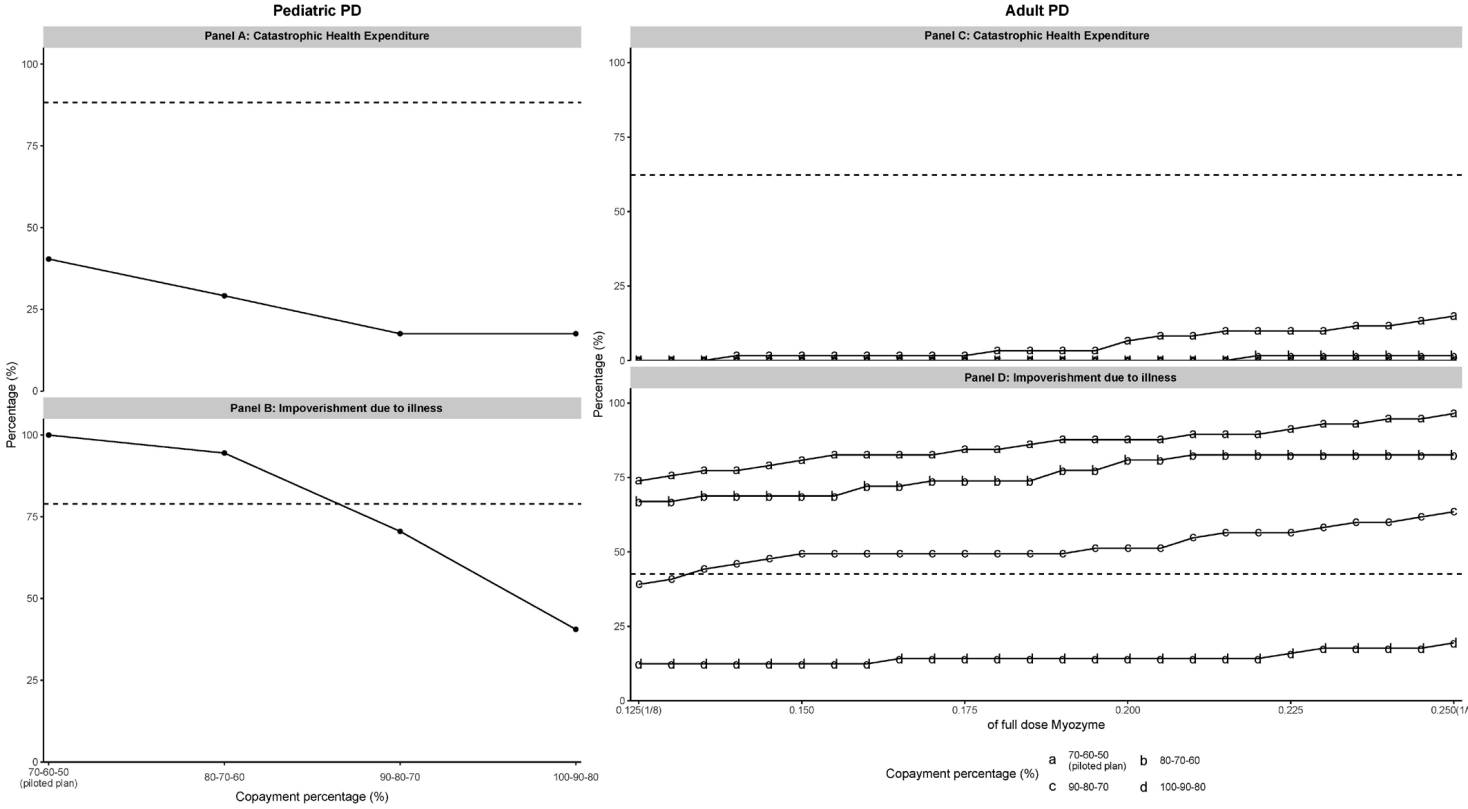
Policy Simulation of the Dosage-Based Model. Includes the simulated results for pediatric PD patients and adult PD patients under a series of reimbursement plans. The “70-60-50” represents a reimbursement plan with three piecewise reimbursements, indicating that patients have a 70% reimbursement ratio for 1-5 vials of Myozyme^®^, 60% reimbursement ratio for vials 6 to 10 of Myozyme^®^, 50% reimbursement ratio for vials 10 to 15 of Myozyme^®^, and no reimbursement for vials 16 and above. The “70-60-50” is the pilot plan, and others are variant plans. For Myozyme^®^, we assumed that pediatric patients would take the full dosage, whereas adult patients would take ⅛ to ¼ of the clinically suggested dosage. Hence for each reimbursement plan, there was one value for pediatric patients (shown in the bar), and several values for adult patients corresponding to each dosage (shown in the line and points). The black dashed line is the rate of CHE or IDI after patients received reimbursement from BMI, shown in Table 3. The reimbursement from BMI is the only reference point because both piloted dosage-based and cost-based models are modified primarily within the scope of BMI.

**Figure 2:**
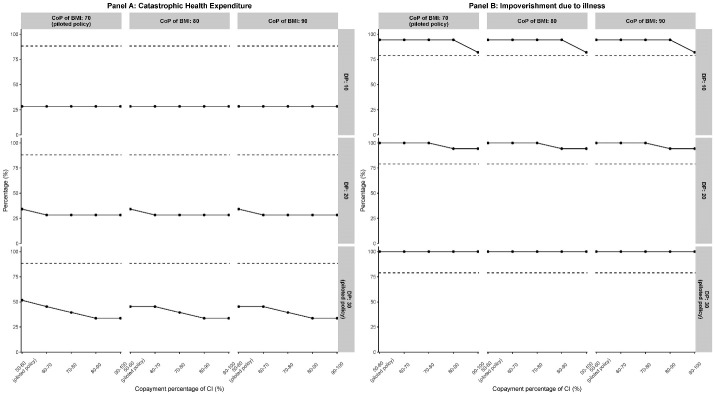
Policy Simulation of the Cost-Based Model (Pediatric Pompe Disease). Includes the simulated results for pediatric PD patients under a series of reimbursement plans. “DP: 30” means the deductible percentage of BMI is 30%. “CoP of BMI: 70” means the co-payment percentage of BMI is 70%. “50-60” represents the co-payment percentage of CI, meaning the part left by basic insurance and more than RMB 12 000 will be covered by the CI with a reimbursement ratio of 50%, and if the annual accumulated amount of the part in the CI is more than RMB 100 000, the reimbursement ratio will increase to 60%. Due to too many parameters in the cost-based model, this figure only presents the simulation results with a fixed ceiling line for BMI and CI. Combination of “DP: 30,” “Cop of BMI: 70,” and “50-60” is the piloted plan, and others are extended plans. For Myozyme^®^, we assumed that pediatric patients would take the full dosage. Hence for each reimbursement plan, there was one value for pediatric patients (shown in the bar). The black dashed line is the rate of CHEs or IDI after patients received reimbursement from BMI, shown in Table 3. The reimbursement from BMI is the only reference point because both piloted dosage-based and cost-based models are modified primarily within the scope of BMI. Abbreviations: BMI, basic medical insurance; CI, commercial insurance; DP, deductible percentage; CoP, co-payment percentage.

**Figure 3:**
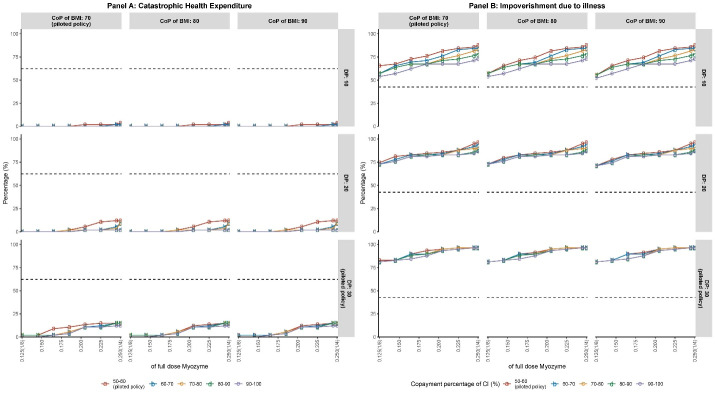
Policy Simulation of the Cost-Based Model (Adult Pompe Disease). Includes the simulated results for pediatric PD patients under a series of reimbursement plans. “DP: 30” means the deductible percentage of BMI is 30%. “CoP of BMI: 70” means the co-payment percentage of BMI is 70%. “50-60” represents the co-payment percentage of CI, meaning the part left by basic insurance and more than RMB 12 000 will be covered by the CI with a reimbursement ratio of 50%, and if the annual accumulated amount of the part in the CI is more than RMB 100 000, the reimbursement ratio will increase to 60%. Due to too many parameters in the cost-based model, this figure only presents the simulation results with a fixed ceiling line for BMI and CI. Combination of “DP: 30,” “Cop of BMI: 70,” and “50-60” is the piloted plan, and others are extended plans. For Myozyme^®^, we assumed that pediatric patients would take the full dosage. Hence for each reimbursement plan, there was one value for pediatric patients (shown in the bar). The black dashed line is the rate of CHEs or IDI after patients received reimbursement from BMI, shown in Table 3. The reimbursement from BMI is the only reference point because both piloted dosage-based and cost-based models are modified primarily within the scope of BMI. Abbreviations: BMI, basic medical insurance; CI, commercial insurance; DP, deductible percentage; CoP, co-payment percentage.

 Under the pilot reimbursement plans, both dosage-based and cost-based models reduced the CHE for both families with pediatric and adult PD patients, and the effect sizes were especially larger for families with adult PD patients (Figures 1 to 3). However, neither model had a significant effect on IDI, and our simulation results even presented a higher IDI than in Table 2.

 The simulated results of the dosage-based model under variant plans indicated that an increased reimbursement rate correlated with a decreasing CHE and IDI (Figure 1). The dosage-based model reached its maximum effect on CHE under the reimbursement rate of “90-80-70” for pediatric patients (Figure 1, Panel A) and “80-70-60” for adult patients (Figure 1, Panel C) (stagnation point or the ceiling of policy), but no stagnation point was found on the effect on IDI (Figure 1, Panel B and D). To explore the possible stagnation point of the dosage-based model on IDI, we extended the simulation to the four piecewise plans (Figure S5). Still, no stagnation point was found, and the four piecewise reimbursement plans did not bring a further reduction on the IDI than the three piecewise reimbursement plans. In summary, under the dosage-based model, both the effects of reducing CHE and IDI were sensitive to the change in reimbursement rate, and the IDI was more so.

 The simulated results of the cost-based model under variant plans showed flat or nearly flats trends, which indicated that the effect of variant plans was almost the same as the pilot policy or more financial input had no additional effects of reducing the CHE and IDI (Figures 2 and 3). The near zero values of CHE compared to the IDI indicated that under the cost-based model, the pilot policy and its variant plans had more obvious effects on CHE than IDI. However, under the cost-based model, the almost flat trends also indicated that the policy seemed to reach the stagnation point more easily. In summary, under the cost-based model, both the effects on reducing CHE and IDI were not sensitive to the change in reimbursement rate. Nevertheless, further results indicated that under the cost-based model, the pediatric PD families were relatively more sensitive to the co-payment percentage of CI than the adult PD families (as shown in Table S1).

 To offer more suggestions for policy improvements, we further simulated the results by combining the advantages of the dosage-based and cost-based models and found that the combination broke through the ceiling effects of two models easily and resulted in reductions for both CHE and IDI (Table S2, Supplementary file 1).

## Discussion

###  Statement of Principal Findings

 Using nationwide cross-sectional data collected in 2018 from China, we evaluated the disease economic burden of PD patients via commonly used indicators: CHE and IDI. Our study highlighted an alarmingly high disease economic burden born by PD patients and their families, which shows that around half the families with PD patients suffered from IDI and about 70% of them suffered from CHE. We further simulated how the above two indicators changed under two reimbursement policies (a dosage-based model and cost-based model) piloted in China. We found that both models reduced the CHE for both families with pediatric and adult PD patients, but they had almost no effect on IDI. Noticeably, the dosage-based model was more sensitive to the change in policy parameters and had more room for improvement.

###  Possible Explanations

 PD patients’ economic burden was notably higher than the average level of the general population in China estimated in 2012, whose ratio of CHE was 12.9%^35^; in contrast, about 70% of the families with PD patients had CHE. PD patients’ economic burden was even higher than that of the patients with nine other rare diseases estimated during 2014 and 2016, including carboxylase deficiency, phenylketonuria, amyotrophic lateral sclerosis, acromegaly, Gaucher’s disease, Duchenne muscular dystrophy, Alport syndrome, Tuberous sclerosis complex, and idiopathic pulmonary fibrosis.^31,36^

 Our results also indicated a high direct non-medical economic burden, accounting for 20–80% of the direct medical economic burden (Table 2). This finding is in line with corresponding evidence, which states that a lack of knowledge, misdiagnoses, and long distance to hospitals are challenges for rare disease patients to access medical service utilization.^37,38^ The number of hospitals visited before finding a definite diagnosis and the low accessibility of PD related information (as shown in Table 2**) **also contributed to this evidence.

 Families with pediatric PD patients face a higher economic burden than families with adult PD patients.The explanation might be that the adults in China usually choose not to utilize healthcare as much to save money for their family but would rather go bankrupt than not treat their children. This explanation can be indirectly supported by higher direct non-medical costs in families with pediatric PD patients (Table 2), which were primarily a result of long-distance travel and long-duration hotel or rental accommodation to pursue quality health services outside of the cities where they live. Another explanation could be a lack of relevant social support in China, which might force parents to spend more time looking after their sick children; thus, depriving them of time for work. Thirdly, it could also be because some adult PD patients were employed and thus could be covered by the Urban Employees Basic Medical Insurance, which is more generous than the Urban Residents’ BMI or the New Rural Cooperative Medical Care, which are the only options for the pediatric PD patients.

 China’s BMI was of almost no help in relieving the economic burden on families with PD patients. The main reason is that the specific medicine for PD, Myozyme^®^, has not yet been included in the National Drug List of BMI. Therefore, PD patients have to pay most costs out of pocket. Although CI is usually considered a supplement of BMI, its function in relieving PD patients’ economic burden was almost zero, which could be a result of the low prevalence of buying CI, and PD medications not being covered by CI in China.

 Our simulation results indicated that both the dosage-based and cost-based reimbursement models could help reduce CHE for both families with pediatric and adult PD patients but had almost no effect on IDI. Unexpectedly, for both families with pediatric PD and adult PD patients, our simulation results presented a higher IDI than reality, which more likely implied an underuse of health services, as our simulation was based on clinical guidelines and only considered the costs related to Myozyme^®^. In addition, related evidence supporting underuse showed that market availability and high economic burden would restrict patients’ access to therapy.^39-41^ Both were identified as problems in the survey we conducted in 2018 in China.

 Our simulation results also indicated that both the dosage-based and cost-based reimbursement models had a risk of causing fairness issues, as families with adult PD patients benefited more from both pilot models compared to families with pediatric PD patients. Although Myozyme^®^ was the only cost-source in our simulation, and the dosage of Myozyme^®^ is based on patients’ weight and adults are heavier than children. However, pediatric PD patients usually took the full dose of Myozyme^®^ and adult PD patients only took ⅛ to ¼ of the Myozyme^®^ dosage, so this more or less cancelled the effects of weight. Therefore, the Myozyme^®^ dosage was not likely to be the reason. Rather, the higher household income of families with adult PD patients compared with families with pediatric PD patients, as shown in Table 2,might provide a clue. Firstly, parents of pediatric PD patients had to spend more time looking after their sick children so they lost time for work; secondly, PD caused physical problems for both children and adults, but adults were still able to work more or less, which was impossible for pediatric PD patients. Despite that, no special supplemental policies have been made to help families with pediatric PD patients.

 Given the advantages and disadvantages of the two models, we also simulated the results by absorbing the advantages of the dosage-based and cost-based models (Figure S6): a combination of the dosage-based model and the CI part of the cost-based model. Namely, patients will undergo the process of a dosage-based model first, and then the rest of the self-burden cost will be covered by CI. This combination may be practical, as CI is a nationally available insurance in China. If the dosage-based model were applied to more places, this model could potentially be implemented at the national level. The results in Figure S6 indicate that the ceiling effects of two models were easily broken through by the combination: the impoverishment of families with pediatric PD patients was reduced significantly, and the difference between families with pediatric PD and adult PD patients was reduced as well. This combination deserves to be considered for places that have more financial resources.

###  Strengths and Limitations

 Using nationwide cross-sectional data, we not only evaluated the disease economic burden of families with PD patients via commonly used indicators, but also simulated how the disease economic burden changed under two pilot reimbursement policies in China. Furthermore, to offer evidence-based suggestions for policy improvement, in addition to the simulation of real-world policies, we also performed a series of simulations based on variant policy parameters. Our study enriches the corresponding evidence related to PD, especially considering that little evidence about the direct cost burden is available, as emphasized by a recent review published in 2018.^42^ This contribution can help other researchers estimate the potential economic benefit of treatment.^43^ Our series of simulations are not only helpful for China to improve its reimbursement policy regarding PD but can also benefit other countries that have been implementing or plan to implement similar policies, as reimbursement policy models that are not based on 100% cost-coverage are commonly used, even in developed countries such as France and Switzerland.^44-46^

 A key limitation of this study is that the PD patients sampled in this study were not covered by the pilot reimbursement policies. Therefore, we can only explore how their economic burden would change by simulating the results assuming they were covered by the pilot policies. This may underestimate the effects of the piloted policies. For instance, the piloted policies may improve the health status of PD patients and further enable themselves or their family to have more opportunities to be employed, and in turn, reduce their family’s economic burden—none of which can be estimated by this study. Another limitation is that we only used data from registered and diagnosed patients. This may underestimate the economic burden of PD in China. Non-registered, especially non-diagnosed patients who were not included, could be poorer because they might be more easily influenced by the barriers to health services. However, this is an international challenge that remains to be solved.^47^ Third, the sample size of PD cases was small and the respondents were recruited by non-probability and convenience sampling methods. The results presented in this study may not be generalizable to all PD patients in China. Fourth, although we collected both direct medical cost and direct non-medical cost data, the use of a self-administrated questionnaire inevitably led to recall-bias. However, the PD patients in China usually had to seek medical care out of their hometown, which thus prevented us from obtaining their medical records from one health information system. Fifth, the two models piloted in China cannot cover all possible schemes used in other cities. Future simulation based on models using international scales is needed. For example, the cost-based model in Latvia is specifically designed for rare diseases,^48^ and is not restricted by the conversion between BMI and CI, as it is in China.

###  Unanswered Questions and Future Research

 The main unanswered question from this work is the influence of indirect costs on the economic burden of families with PD patients. Indirect cost may be an important explanation for why adult PD patients benefit more from the simulated policy, as we discussed earlier. As previous studies have found, the indirect costs also constitute a significant part of the patients’ burden,^4^ and related evidence is limited^42^. Particularly, in the case of pediatric patients, parents usually put more time and energy as a caregiver and could suffer more from a loss of productivity. Another unanswered question is the evidence on IOPD. Infant onset PD patients barely survive within the first year of life. But evidence indicates that the life expectancy of IOPD is much improved if Myozyme^®^ is applied early.^49^ Therefore, IOPD could benefit more from Myozyme^®^, and corresponding evidence is more substantial to promote the inclusion of Myozyme^®^ in reimbursement lists.

## Conclusion

 Families with PD patients in China faced a notably high economic burden from direct medical costs and direct non-medical costs. Families with pediatric PD patients had a higher economic burden than families with adult PD patients. China’s BMI was almost no help in relieving the economic burden on families with PD patients. Both the dosage-based and cost-based reimbursement models could reduce the CHE for both families with pediatric and adult PD patients but had almost no effect on IDI. The dosage-based and cost-based reimbursement models could benefit families with adult PD patients more, which implies an inequality caused by a unified policy regardless of the actual needs of different types of patients. Our study provides corresponding evidence for other countries who try to improve their policies to reduce the disease burden caused by rare diseases.

## Acknowledgements

 The authors would like to thank China Pompe Care Center and the Illness Challenge Foundation for their assistance in distributing the survey questionnaire.

## Ethical issues

 The study was approved by the Committee on the Use of Human and Animal Subjects in Teaching and Research, Hong Kong Baptist University (HASC no: FRG2/15-16/052). All participants provided informed consent before the survey was conducted.

## Competing interests

 Authors declare that they have no competing interests.

## Authors’ contributions

 DD developed the survey questionnaire, collected the data, and conducted a preliminary analysis. SC conducted the full data analysis and wrote the initial draft of the manuscript. All authors contributed to the final version of the manuscript. All authors have read and approved the final manuscript.

## Funding

 The study was supported by the Faculty Research Grant, Hong Kong Baptist University (No: FRG2/15-16/052) and the Rare Disease Research Grant funded by The Illness Challenge Foundation.

## Supplementary files

Supplementary file 1 contains Figures S1-S6 and Tables S1-S2.
