# Supplementary material for: Improving Insurance Protection for Rare Diseases: Economic Burden and Policy Effects — Simulation of People With Pompe Disease in China
Source: Int J Health Policy Manag. 2022 Oct 24;12:6282. doi: 10.34172/ijhpm.2022.6282 (PMC10125180; doi:10.34172/ijhpm.2022.6282)
Supplement: Supplementary file 1 — contains Figures S1-S6 and Tables S1-S2. [file ijhpm-12-6282-s001.pdf]

Article title: Improving Insurance Protection for Rare Diseases: Economic Burden and Policy Effects — Simulation of People With Pompe Disease in China

Journal name: International Journal of Health Policy and Management (IJHPM)

Authors' information: Shanquan Chen[1], Dong Dong[2,3]*

[1]School of Clinical Medicine, University of Cambridge, Cambridge, UK.

[2]Jockey Club School of Public Health and Primary Care, Chinese University of Hong Kong, Hong Kong, China.

[3]Shenzhen Research Institute, The Chinese University of Hong Kong, Shenzhen, China.

(*Corresponding author: dongdong@cuhk.edu.hk)

Supplementary file 1

S-Fig. 1 Bottom-up approach in estimation of household income

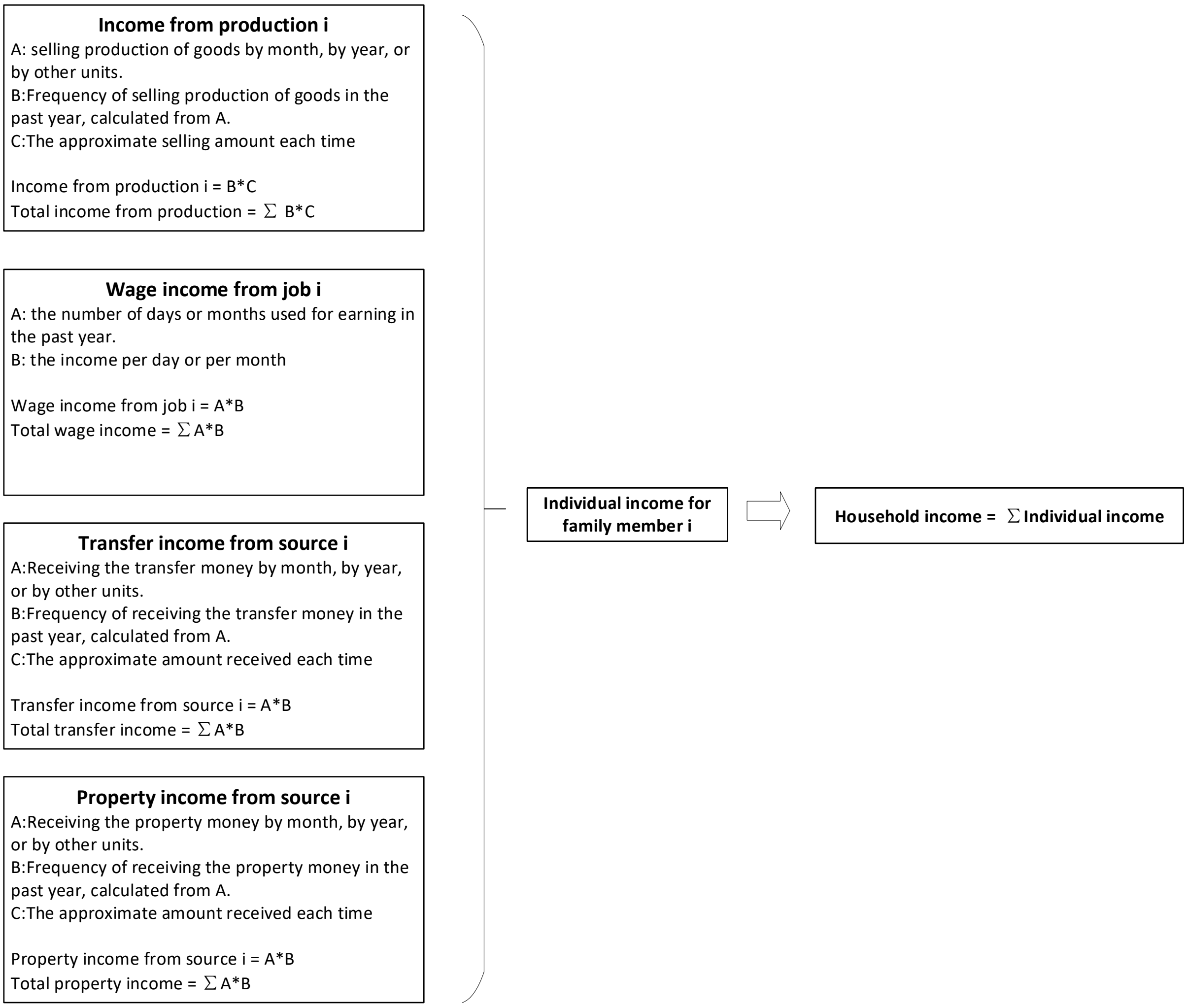

S-Fig.2 Bottom-up approach in estimation of outpatient expenses

Outpatient expenses

A1: How often do you visit outpatient in primary care?
B1: The approximate cost for each outpatient visit in primary care.
C1: How much reimbursement you can get from basic medical insurance?
D1: How much reimbursement you can get from commercial insurance?
E1: How did you get there, on foot, by bike, by car, or by bus?
F1: How many people companied you get there? Then plus 1 patient.
G1.1: If by bus, what is the ticket price of the bus to get there?
G1.2: if by car, what is the cost of the gas or electric to get there?
H1.1: Do you need to eat outside when visiting the outpatient in primary care, if so, how much is the cost per person?
H1.2: What is the frequency of eating outside when you visit outpatient in primary care?
I1.1: Do you need to spend the night outside when you visit outpatient in primary care, if so, how much is the cost per person?
I1.2: How many nights do you need to spend outside?

A2: How often do you visit outpatient in secondary care?
B2: The approximate cost for each outpatient visit in secondary care.
C2: How much reimbursement you can get from basic medical insurance?
D2: How much reimbursement you can get from commercial insurance?
E2: How did you get there, on foot, by bike, by car, or by bus?
F2: How many people companied you get there? Then plus 1 patient.
G2.1: If by bus, what is the ticket price of the bus to get there?
G2.2: if by car, what is the cost of the gas or electric to get there?
H2.1: Do you need to eat outside when visiting the outpatient in secondary care, if so, how much is the cost per person?
H2.2: What is the frequency of eating outside when you visit outpatient in secondary care?
I2.1: Do you need to spend the night outside when you visit outpatient in secondary care, if so, how much is the cost per person?
I2.2: How many nights do you need to spend outside?

A3: How often do you visit outpatient in tertiary care?
B3: The approximate cost for each outpatient visit in tertiary care.
C3: How much reimbursement you can get from basic medical insurance?
D3: How much reimbursement you can get from commercial insurance?
E3: How did you get there, on foot, by bike, by car, or by bus?
F3: How many people companied you get there? Then plus 1 patient.
G3.1: If by bus, what is the ticket price of the bus to get there?
G3.2: if by car, what is the cost of the gas or electric to get there?
H3.1: Do you need to eat outside when visiting the outpatient in primary care, if so, how much is the cost per person?
H3.2: What is the frequency of eating outside when you visit outpatient in tertiary care?
I3.1: Do you need to spend the night outside when you visit outpatient in tertiary care, if so, how much is the cost per person?
I3.2: How many nights do you need to spend outside?

Outpatient expenses = A1*B1 + A2*B2 + A3*B3
Reimbursement from basic medical insurance = A1*C1 + A2*C2 + A3*C3
Reimbursement from commercial insurance = A1*D1 + A2*D2 + A3*D3
Direct non-medical expenses = (F1*G1.1*2 or G1.2*2 + F1*H1.1*H1.2 + F1*I1.1*I1.2) + (F2*G2.1*2 or G2.2*2 + F2*H2.1*H2.2 + F2*I2.1*I2.2) + (F3*G3.1*2 or G3.2*2 + F3*H3.1*H3.2 + F3*I3.1*I3.2)

S-Fig.3 Bottom-up approach in estimation of hospitalization expenses

Hospitalization expenses

A1: How many times were you admitted to the inpatient in the past year.
B1: For the first time, where you were admitted to the inpatient.
C1: How much did you spend on this time of admission?
D1: At this time of admission, how much reimbursement do you get from basic medical insurance?
E1: At this time of admission, how much reimbursement you can get from commercial insurance?
F1: How did you get there, on foot, by bike, by car, or by bus?
G1: How many people companied you get there?
H1.1: If by bus, what is the ticket price of the bus to get there?
H1.2: if by car, what is the cost of the gas or electric to get there?
I1: How many days have you been hospitalized?
J1: What's the average amount of money spent on food per person per day?
K1: What's the average amount of money spent on accommodation per person per day

B2: For the second time, where you were admitted to the inpatient.
C2: How much did you spend on this time of admission?
D2: At this time of admission, how much reimbursement do you get from basic medical insurance?
E2: At this time of admission, how much reimbursement you can get from commercial insurance?
F2: How did you get there, on foot, by bike, by car, or by bus?
G2: How many people companied you get there?
H2.1: If by bus, what is the ticket price of the bus to get there?
H2.2: if by car, what is the cost of the gas or electric to get there?
I2: How many days have you been hospitalized?
J2: What's the average amount of money spent on food per person per day?
K2: What's the average amount of money spent on accommodation per person per day

....

inpatient expenses = C1 + C2 + ...
Reimbursement from basic medical insurance = D1 + D2 + ...
Reimbursement from commercial insurance = E1 + E2 + ...
Direct non-medical expenses = [(G1+1)*H1.1*2 or H1.2*2 + (G1+1)*I1*J1 + G1*I1*K1] + [(G2+1)*H2.1*2 or H2.2*2 + (G2+1)*I2*J2 + G2*I2*K2] + ...

S-Fig.4 Bottom-up approach in estimation of self-purchased drugs or equipment costs

Self-purchased drugs or equipment costs

A1: How often do you self-purchase drugs or equipment in your town in the past year?
B1: The approximate cost for each time.
C1: How did you get there, on foot, by bike, by car, or by bus?
D1: How many people companied you get there? Then plus 1 patient.
E1.1: If by bus, what is the ticket price of the bus to get there?
E1.2: if by car, what is the cost of the gas or electric to get there?
F1.1: Do you need to eat outside when self-purchasing drugs or equipment in your town, if so, how much is the cost per person?
F1.2: What is the frequency of eating outside when you self-purchase drugs or equipment in your town?
G1.1: Do you need to spend the night outside when you self-purchase drugs or equipment in your town, if so, how much is the cost per person?
G1.2: How many nights do you need to spend outside?

A2: How often do you self-purchase drugs or equipment in your county in the past year?
B2: The approximate cost for each time.
C2: How did you get there, on foot, by bike, by car, or by bus?
D2: How many people companied you get there? Then plus 1 patient.
E2.1: If by bus, what is the ticket price of the bus to get there?
E2.2: if by car, what is the cost of the gas or electric to get there?
F2.1: Do you need to eat outside when self-purchasing drugs or equipment in your town, if so, how much is the cost per person?
F2.2: What is the frequency of eating outside when you self-purchase drugs or equipment in your town?
G2.1: Do you need to spend the night outside when you self-purchase drugs or equipment in your town, if so, how much is the cost per person?
G2.2: How many nights do you need to spend outside?

A3: How often do you self-purchase drugs or equipment in your city in the past year?
B3: The approximate cost for each time.
C3: How did you get there, on foot, by bike, by car, or by bus?
D3: How many people companied you get there? Then plus 1 patient.
E3.1: If by bus, what is the ticket price of the bus to get there?
E3.2: if by car, what is the cost of the gas or electric to get there?
F3.1: Do you need to eat outside when self-purchasing drugs or equipment in your town, if so, how much is the cost per person?
F3.2: What is the frequency of eating outside when you self-purchase drugs or equipment in your town?
G3.1: Do you need to spend the night outside when you self-purchase drugs or equipment in your town, if so, how much is the cost per person?
G3.2: How many nights do you need to spend outside?

A4: How often do you self-purchase drugs or equipment in other cities in the past year?
B4: The approximate cost for each time.
C4: How did you get there, on foot, by bike, by car, or by bus?
D4: How many people companied you get there? Then plus 1 patient.
E4.1: If by bus, what is the ticket price of the bus to get there?
E4.2: if by car, what is the cost of the gas or electric to get there?
F4.1: Do you need to eat outside when self-purchasing drugs or equipment in your town, if so, how much is the cost per person?
F4.2: What is the frequency of eating outside when you self-purchase drugs or equipment in your town?
G4.1: Do you need to spend the night outside when you self-purchase drugs or equipment in your town, if so, how much is the cost per person?
G4.2: How many nights do you need to spend outside?

Self-purchased drugs or equipment costs = A1*B1 + A2*B2 + A3*B3 + A4*B4
Direct non-medical expenses = (D1*E1.1*2 or E1.2*2 + D1*F1.1*F1.2 + D1*G1.1*G1.2) + (D2*E2.1*2 or E2.2*2 + D2*F2.1*F2.2 + D2*G2.1*G2.2) + (D3*E3.1*2 or E3.2*2 + D3*F3.1*F3.2 + D3*G3.1*G3.2) + (D4*E4.1*2 or E4.2*2 + D4*F4.1*F4.2 + D4*G4.1*G4.2)

S-Fig. 5 Policy simulation of the dosage-based model with four piecewise reimbursement

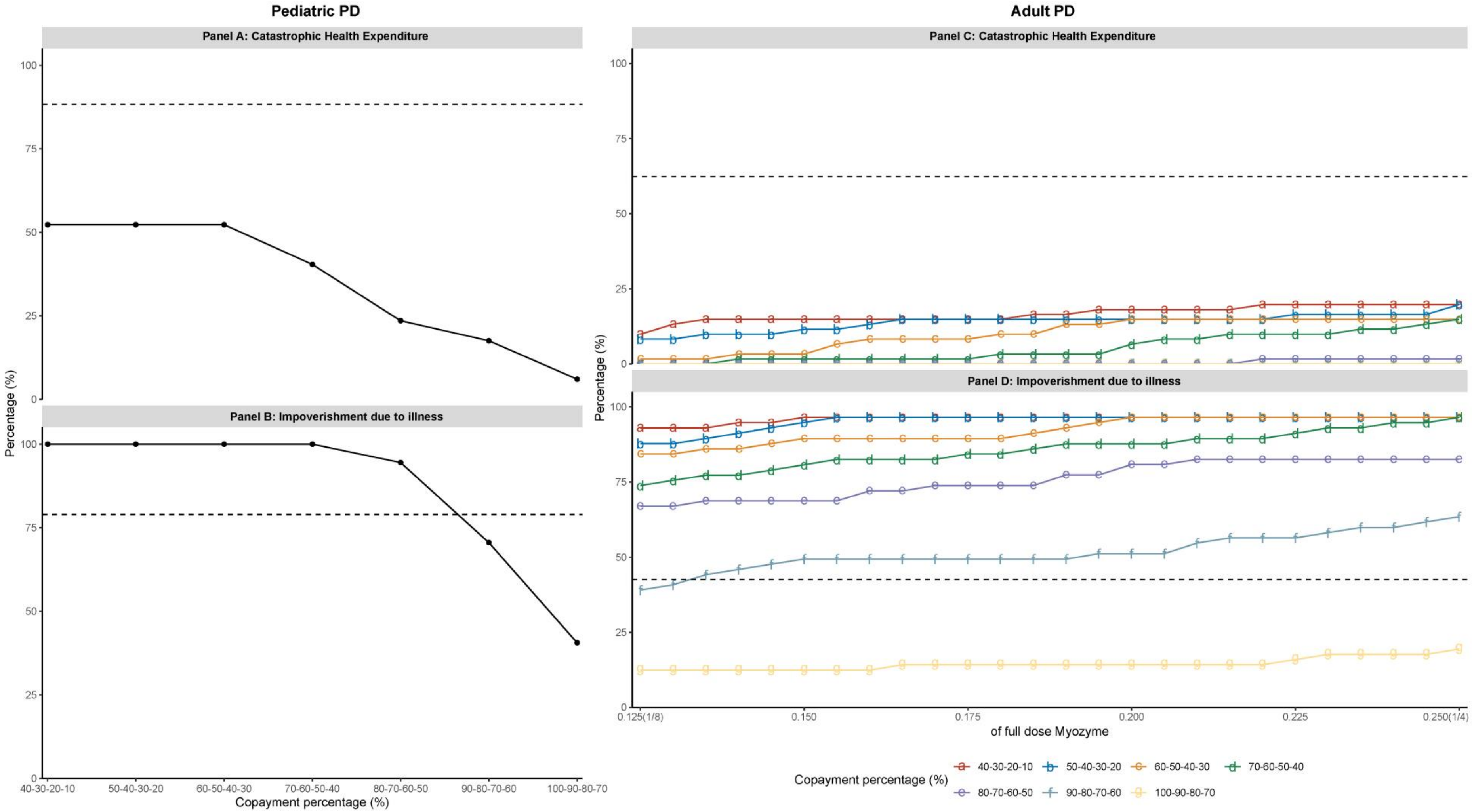

Panel A presents the simulated results for pediatric PD patients, and panel B presents the simulated results for adult PD patients, under a series of reimbursement plans. The "40-30-20-10" represents a reimbursement plan with four piecewise reimbursement, indicating that patients have a 40% reimbursement ratio for 1-5 vials of Myozyme®, 30% reimbursement ratio for vials 6 to

10 of Myozyme®, 20% reimbursement ratio for vials 10 to 15 of Myozyme®, 10% reimbursement ratio for vials 16 to 20 of Myozyme®, and no reimbursement for vials 21 and above. For Myozyme®, we assume that pediatric patients take the full clinically suggested dosage, whereas adult patients take ⅛ to ¼ of the clinic suggested dosage. So for each reimbursement plan, there is one value for pediatric patients (shown in the bar), but several values for adult patients corresponding to each dosage (shown in line and points). The black dashed line is the rate of catastrophic health expenditure (CHE) or impoverishment due to illness after patients received reimbursement from basic medical insurance, shown in Table 3. We only choose the reimbursement from basic medical insurance as reference because both the piloted dosage-based and cost-based models are reformed primarily within the scope of basic medical insurance.

S-Fig. 6 Policy simulation of combination of the dosage-based model and CI part of the cost-based model

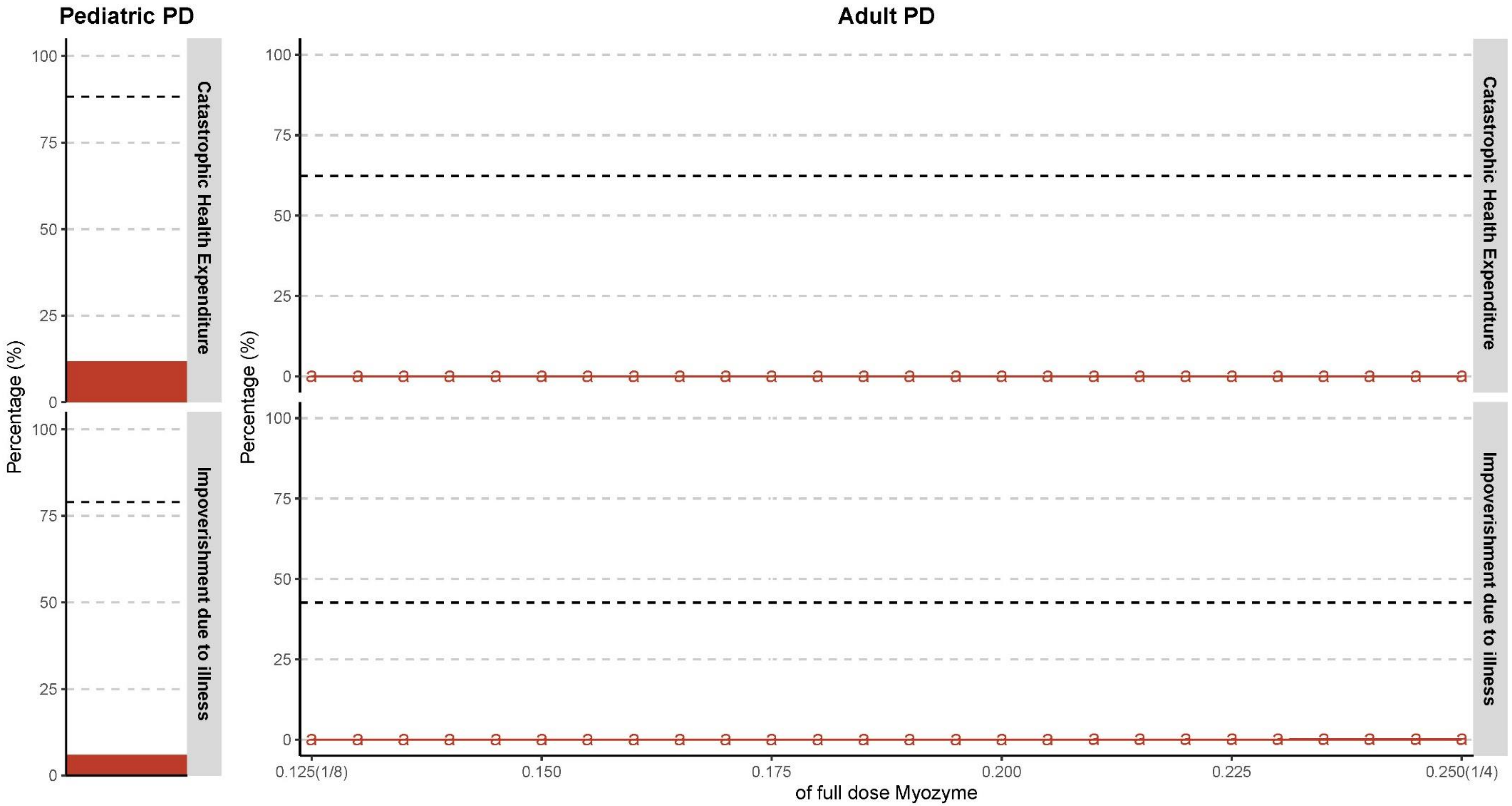

In this simulation, patients will benefit from the dosage-based model first, the rest of self-burden cost will be covered by CI. Panel A presents the simulated results for pediatric PD patients, and panel B presents the simulated results for adult PD patients. For Myozyme®, we assume that pediatric patients take the full clinic suggested dosage, whereas adult patients take ⅛ to ¼ of the clinic suggested dosage. So for each reimbursement plan, there is only one value for pediatric patients (shown in the bar), but several values for adult patients corresponding to each dosage (shown in

line and points). This simulation only simulates the piloted policy parameters, so only one bar or one line. The black dash line is the rate of catastrophic health expenditure (CHE) or impoverishment due to illness after patients received reimbursement from basic medical insurance, shown in table 3. We only choose the reimbursement from basic medical insurance as references because both piloted dosage-based and cost-based models are reformed primarily within the scope of basic medical insurance. CI = commercial insurance.

S-Table 1 Sensitive of catastrophic health expenditure and impoverishment on different policy parameters in cost-based model

	Catastrophic health expenditure				Impoverishment			
	Coefficient	Standardized coefficient	se	P–value	Coefficient	Standardized coefficient	se	P–value
**Pediatric PD patients**								
(Intercept)	53.6	-	0.71	0.0000	102.5	-	0.47	0.0000
Deductible percentage (per 10%)	8.2	0.73	0.06	0.0000	4.8	0.75	0.04	0.0000
Co-payment percentage of basic medical insurance (per 10%)	–0.1	-0.02	0.04	0.0023	–0.4	-0.08	0.03	0.0000
Co-payment percentage of CI (per 10%)	–3.2	-0.48	0.03	0.0000	–1.4	-0.39	0.02	0.0000
Ceiling of BMI (per 10,000)	–0.4	-0.09	0.02	0.0000	–0.1	-0.05	0.01	0.0000
Ceiling of CI (1) (per 10,000)	–0.4	-0.15	0.01	0.0000	–0.1	-0.05	0.01	0.0000
Ceiling of CI (2) (per 10,000)	–0.1	-0.10	0.01	0.0000	0.0	0.00	0.00	1.0000
**Adult PD patients**								
(Intercept)	–9.8	-	0.17	0.0000	50.4	-	0.19	0.0000
Deductible percentage (per 10%)	3.6	0.62	0.01	0.0000	10.5	0.75	0.01	0.0000
Co-payment percentage of basic medical insurance (per 10%)	–0.1	-0.02	0.01	0.0000	–0.9	-0.09	0.01	0.0000
Co-payment percentage of CI (per 10%)	–0.5	-0.14	0.01	0.0000	–1.2	-0.15	0.01	0.0000
Ceiling of BMI (per 10,000)	–0.1	-0.03	0.01	0.0000	–0.2	-0.04	0.01	0.0000
Ceiling of CI (1) (per 10,000)	0.0	-0.01	0.00	0.0089	0.0	-0.01	0.00	0.0000
Ceiling of CI (2) (per 10,000)	0.0	0.00	0.00	1.0000	0.0	0.00	0.00	1.0000
% of full dose Myozyme®	7.1	0,52	0.03	0.0000	19.0	0.58	0.03	0.0000

† BMI, basic medical insurance; CI, commercial insurance.

‡These results come from a series of linear regressions, with the rate of catastrophic health expenditure (CHE) or impoverishment due to illness as the dependent variable, and policy parameters as the independent variables. Data comes from our policy simulations (supplemented). To make the coefficients of different policy parameters comparable, policy parameters taking the unit usually changed. For instance, in practice in China, deductible percentages usually changed incrementally by 10 percentage points, namely, increasing by 10%, 20%, etc. Sensitivity is measured by the absolute value of the coefficients; the higher the coefficient is, the higher the sensitivity is. For example, for CHE, this table indicated that it is more sensitive to the change in the

Deductible percentage (8.2), followed by the Co-payment percentage of CI (3.2). Other settings can also use this table. According to the properties of linear regression, the coefficient in this table can be multiplied or divided (e.g., if one country usually changes the deductible percentage by 5 percentage points, then its coefficient will be 8.2/2 = 4.1).

S-Table 2 Policy simulation of combination of the dosage-based model and CI part of the cost-based model

	Percentage of catastrophic health expenditure (%)	Percentage of impoverishment due to illness (%)
**Pediatric PD patients**		
Full clinically suggested dosage	11.8	6.0
**Adult PD patients**		
⅛ to ¼ of the clinically suggested dosage		
0.125 (1/8)	0.0	0.0
0.15	0.0	0.0
0.175	0.0	0.0
0.2	0.0	0.0
0.225	0.0	0.0
0.25 (1/4)	0.0	0.1

In this simulation, patients benefit from the dosage-based model first, the rest of self-burden cost is covered by CI. For Myozyme®, we assumed that pediatric patients take the full clinically suggested dosage, and adult patients take ⅛ to ¼ of the clinically suggested dosage. So for each reimbursement plan, there was one value for pediatric patients, but several values for adult patients corresponding to each dosage. This simulation only simulated the piloted policy parameters. CI is commercial insurance.
